# Novel Ultrasound-Guided Radiofrequency Ablation of the Epicondylar Branch of the Posterior Cutaneous Nerve of the Forearm for Recalcitrant Lateral Epicondylosis

**DOI:** 10.7759/cureus.61222

**Published:** 2024-05-28

**Authors:** Siddardth Umapathy, Matthew Miller, Yin-Ting Chen

**Affiliations:** 1 Department of Orthopaedics and Rehabilitation, Walter Reed National Military Medical Center, Bethesda, USA

**Keywords:** epicondylosis, epicondylopathy, lateral epicondylitis, radiofrequency ablation, elbow pain, interventional, ultrasonography

## Abstract

This report describes a novel technique for the treatment of recalcitrant lateral epicondylosis (LE) by radiofrequency ablation (RFA) of the epicondylar branch of the posterior cutaneous nerve of the forearm (PCNF-BrEpi). Here, we describe two patients suffering from recalcitrant LE who were treated with ultrasound-guided RFA of the PCNF-BrEpi in the outpatient pain clinic setting. Patient follow-up was made at eight weeks, five months, and seven months. Numerical pain rating (NPR) for pain and Upper Extremity Functional Index-15 (UEFI-15) were obtained at baseline and at each of the follow-ups. Both patients reported significant improvement in their pain and function quickly. RFA may be a viable treatment option for recalcitrant LE. Larger comparative trials and further investigation are needed to establish results in comparison to conventional treatments and to validate RFA as a treatment option in recalcitrant LE.

## Introduction

Lateral epicondylosis (LE) is a common musculoskeletal condition. LE is a chronic tendinopathy of the common extensor tendon (the major attachment point for extensor muscles of the forearm originating at the lateral humeral epicondyle) characterized by the chronic degeneration of the tendon tissue and the development of disorganized collagen fibers with or without the infiltration of neovascularization and pain fibers [1,2]. The worldwide prevalence of LE is estimated to impact 1-3% of the general population [3]. The common complaint of patients afflicted with LE is the persistent pain over the lateral epicondyle region worsened by gripping, wrist extension, and direct pressure. Although most LE is commonly self-limiting (6-9 months duration), some do not respond to treatment and become recalcitrant, and symptoms can persist for 18 months to two years and in some cases even longer [2,4]. The term "chronic lateral epicondylitis" is also commonly used in the literature though it does not accurately reflect the presence of tendinopathy. Most LE will respond well to conservative treatments such as rest, nonsteroidal anti-inflammatory drugs (NSAIDs), counterforce orthosis, and physical and occupational therapy [1]. Steroid injection treatment is also common, though a recent study has shown that it may have a negative effect in the intermediate term [5]. Interventional treatments are indicated if conservative treatments fail. Treatments targeting tendinopathic lesions such as extracorporeal shockwave therapy, Tenex, and laser therapy have demonstrated benefit [3,6]. Similarly, orthobiological treatments such as autologous blood, prolotherapy, and platelet-rich plasma (PRP) are also helpful [7,8]. Surgical tenotomies are generally reserved for severe cases [9]. Despite these treatments, a small percentage of patients with LE do not respond to the treatment and may develop chronic recalcitrant LE.

The lateral epicondyle is unique because it is an osteotendinous structure that has a known dedicated terminal sensory nerve. The lateral epicondyle is innervated by the epicondylar branch of the posterior cutaneous nerve of the forearm (PCNF-BrEpi), which was first described in 1948 by Gardner [10]. The PCNF branches from the radial nerve as the radial nerve exits the spiral groove at the mid-shaft of the posterolateral humerus. The PCNF divides into anterior and posterior divisions by the distal third of the humerus, and then the posterior division gives rise to the PCNF-BrEpi, as well as a potential motor branch to the anconeus (PCNF-BrA) described by Maida et al. [11]. The ultrasound (US) visualization and accurate US-guided injection were validated by Maida et al. in 2017 [11]. Surgical denervation of the PCNF-BrEpi has shown to be helpful for chronic recalcitrant LE [1], but to date, there are no reports of non-surgical interventional treatments targeting the PCNF-BrEpi for the management of chronic recalcitrant LE. Here, we describe a novel technique we developed to perform radiofrequency ablation (RFA) targeting the PCNF-BrEpi for the treatment of chronic recalcitrant LE.

## Case presentation

US visualization of the PCNF-BrEpi

**Figure 1:**
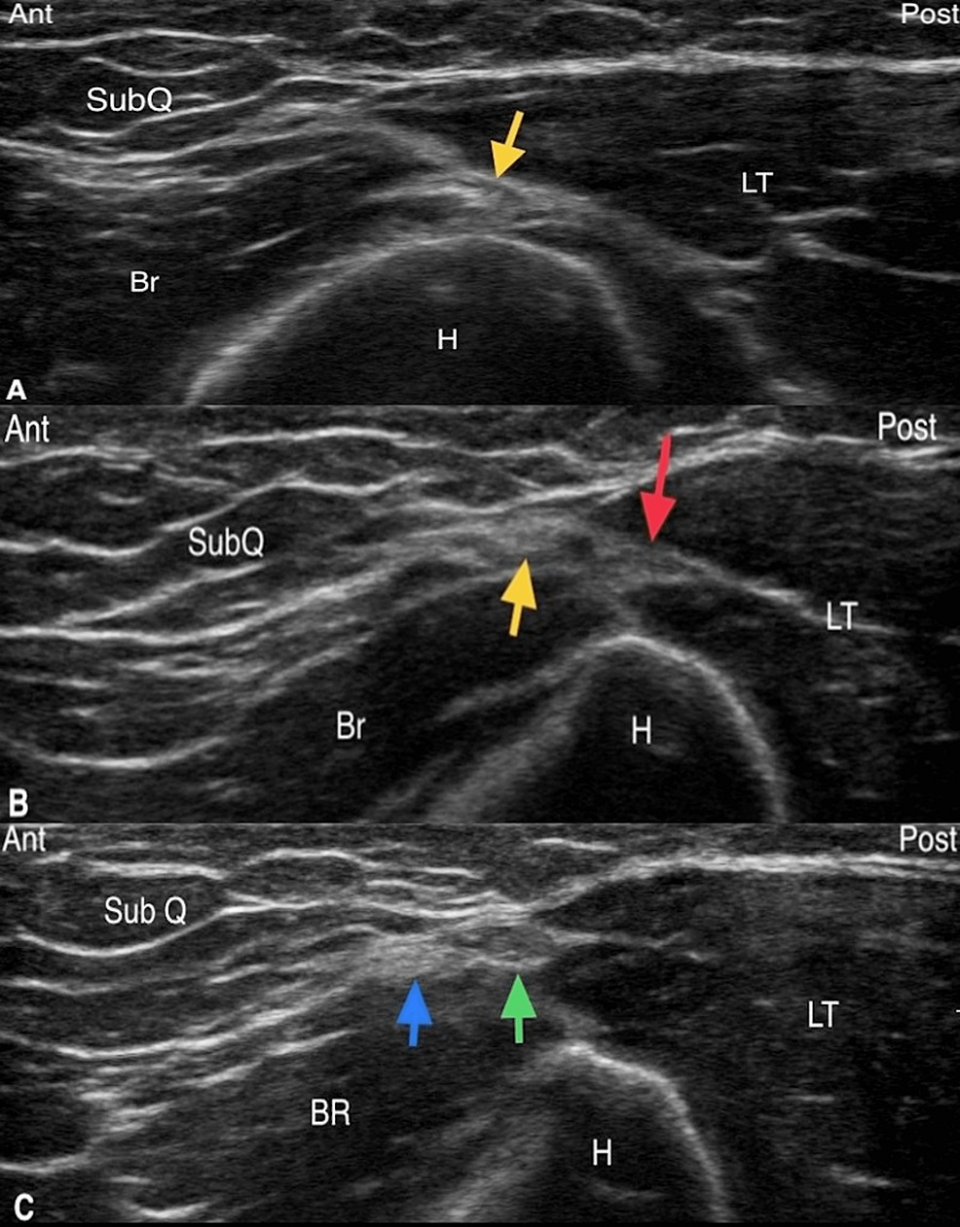
(A) Sonographic identification, in the transverse axis view, of the radial nerve (yellow arrow) at the level of the spiral groove at the lateral posterolateral humerus. (B) Transverse axis view depicting the PCNF (red arrow) arising as the posterior division of the radial nerve (yellow arrow) upon exiting the spiral groove. (C) By the distal third of the humerus, the transverse view at the lateral brachium shows the PCNF dividing into an anterior division (blue arrow) and a posterior division (green arrow) within the LT-BR interval. SubQ: subcutaneous tissue; Br: brachialis; LT: long head of the triceps; H: humerus; BR: brachioradialis; Ant: anterior; Post: posterior; PCNF: posterior cutaneous nerve of the forearm; LT-BR: lateral triceps-brachioradialis

The visualization of PCNF-BrEpi is performed as described by Maida and colleagues [11]. The transverse axis view at the level of the spiral groove at the posterolateral humerus visualizes the radial nerve emerging in the fascial plane dividing the anterior and the posterior compartments (Figure 1A), which then quickly divides into the radial nerve proper and the PCNF (Figure 1B) upon exiting the spiral groove. The radial nerve enters the brachioradialis and the PCNF enters the triceps posterior to the lateral intermuscular septum. By the distal third of the humerus, the transverse axis view at the lateral brachium shows the PCNF dividing into an anterior division and a posterior division within the lateral triceps-brachioradialis (LT-BR) interval, and both enter the subcutaneous plane (Figure 1C). The posterior division eventually reaches the lateral epicondyle bony surface as the PCNF-BrEpi. The PCNF-BrEpi is often accompanied by a small blood vessel on its posterior aspect.

Illustrative cases and RFA technique

The first patient was a 35-year-old male United States Army soldier with a seven-year history of bilateral chronic recalcitrant LE who failed appropriate conservative managements (activity modification, PT, and oral medications (NSAIDs)) and interventional treatments to include dehydrated human amnion/chorion membrane allograft injections and surgical debridement. His MRI demonstrated chronic thickening of bilateral common extensor tendons. The second patient was a 38-year-old male who was involved in a parachuting accident leading to right elbow lateral collateral ligament and ulnar collateral ligament tear status post lateral collateral ligament repair, with chronic right LE, who also failed conservative management (activity modification, PT, and oral medications (NSAIDs)). The patient was considering a possible surgical revision, but his hand surgeon recommended against the surgery.

**Figure 2:**
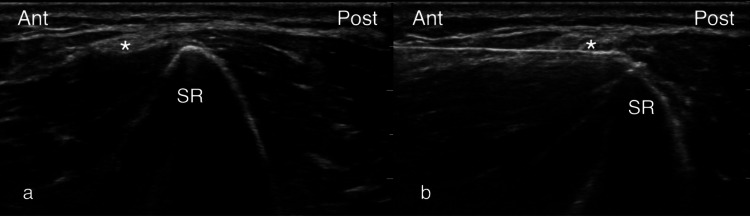
The placement of RF probe. (A) Sonographic transverse axis view at the level proximal to the lateral epicondyle, at which the contour of the humerus assumes a peak appearance at the SR. The PCNF-BrEpi (asterisk, *) is seen as a small caliber with several fascicles, located near the peak of the SR. (B) The RF probe is guided in-plane, in an anterior-to-posterior direction, to contact the undersurface of the PCNF-BrEpi. Ant: anterior; Post: posterior; RF: radiofrequency; SR: supracondylar ridge; PCNF-BrEpi: epicondylar branch of the posterior cutaneous nerve of the forearm

The procedure was performed with the patient placed in a supine position with the shoulder in 90 degrees abduction and 90 degrees external rotation, the elbow in 90 degrees flexion, and the forearm in supination. PCNF-BrEpi was visualized in accordance with the technique described above. A prognostic block under US guidance was first performed. Sterile preparation of the skin was performed, followed by intracutaneous infiltration with 1 mL of 1% lidocaine for local anesthesia. The PCNF-BrEpi was located at about 1-3 cm proximal to the lateral epicondyle where the lateral supraepicondylar ridge maintained a sharp peak (Figure 2A). The PCNF-BrA was not visualized in either patient. A 25-gauge 2-inch needle entered the skin of the anterior distal arm at about 1-2 cm proximal to the elbow crease and was guided in an in-plane, anterior-to-posterior direction until reaching the PCNF-BrEpi, followed by the injection 0.5 mL of 2% lidocaine. RFA was subsequently performed after the patients reported at least 50% pain relief after the block. The PCNF-BrEpi visualization and RF probe approach were performed as described above. The PCNF-BrEpi was injected with 1 mL of 4% lidocaine prior to the RFA. A 22-gauge 50-mm radiofrequency (RF) probe was placed on the undersurface of the PCNF-BrEpi under direct US visualization to ensure maximum contact with the active tip (Figure 2B). Sensory testing was positive at <0.5 V at 50 Hz, and motor testing was negative at 1.5 V at 2 Hz. The RFA was performed (RFP-100A RF Puncture Generator, Baylis Medical, Montréal, Quebec, Canada) at one cycle of 85 degrees Celsius for 90 seconds, followed by the injection of 0.5 mL of 2% lidocaine, 0.5 mL of 0.75% bupivacaine, and 10 mg of dexamethasone. Neither patient experienced any complications during or after the procedures.

Patient follow-up was made at eight weeks, five months, and seven months. Numerical pain rating (NPR) for pain and Upper Extremity Functional Index-15 (UEFI-15) were obtained at baseline and at each of the follow-ups (Table 1). Both patients reported significant improvement in their pain and function quickly. By eight weeks post-procedure, both patients were able to return to normal upper extremity activities at near-full strength with minimal functional limitations. Patient 1 was able to return to duty with the complete function required of him as an infantryman. Patient 2 experienced minimal residual discomfort during activities but did not interfere with his function.

**Table 1:** The NPR score and UEFI-15 at baseline and at each follow-up. Higher percentage=higher score on UEFI-15=increased functionality. UEFI-15 MCID=6.7%. MDC=8.8%. MCID: minimal clinically important difference; MDC: minimum detectable change; NPR: numeric pain rating; UEFI-15: Upper Extremity Functional Index-15

		Baseline	Diagnostic block	Eight weeks	Five months	Seven months
Patient 1	NPR	7/10	3/10	0/10	0/10	4/10
	UEFI-15 (%)	53.3%		95%	100%	100%
Patient 2	NPR	5/10	0/10	2/10	2/10	2/10
	UEFI-15 (%)	73.3%		90%	83.3%	81.7%

## Discussion

There are several technical factors involved in this technique. First, the PCNF-BrEpi is a relatively superficial nerve; therefore, care must be taken to ensure the cutaneous layer is not injured during thermocoagulative treatments. If there is an adequate subcutaneous layer, distance from the skin can be increased by placing the RF probe deep into the PCNF-BrEpi. Another option is to place the RF probe superficial to the PCNF-BrEpi and then press the nerve down with the RF probe to increase the distance from the cutaneous tissue. Easing the downward pressure of the US transducer over the treatment site to minimize tissue compression will also increase the RF probe distance from the cutaneous tissue. Second, because PCNF-BrEpi is a small-caliber nerve, its similar echotexture may be difficult to visually distinguish and isolate from the surrounding subcutaneous tissue, and this effect becomes more noticeable closer to the epicondyle with the thinning of the subcutaneous tissues. Several modifications can improve the accurate isolation of the PCNF-BrEpi. Firstly, RFA should be performed at a few centimeters proximal to the epicondyle where the local soft tissue is plentiful and can be easily discerned from the PCNF-BrEpi. The bony contour has a "mountain peak" appearance at this level, as seen in Figure 2A, which can serve as an obvious landmark; distal to this site, the bony contour flattens out, and the PCNF-BrEpi starts to become harder to distinguish from the surrounding tissue. Secondly, sensory testing prior to the RFA can help to ascertain contacting the nerve. Thirdly, a low-volume perineural injection can accentuate the nerve from its surrounding tissue through the enhancement of the epineurium.

The neurovasculature anatomy near the PCNF-BrEpi deserves some consideration. Consistent with the report by Maida and colleagues [11], we found a small-caliber vessel accompanying the PCNF-BrEpi at the distal arm and near the epicondyle. Care can be taken to increase the distance from the vessel during RFA, but the proximity of the vessel to the PCNF-BrEpi makes injury likely unavoidable. Vascular injury should be avoided if possible, but given that the vessel is small, that it's not a known vascular supply for any anatomical structures, and that its course is cutaneous, its injury during RFA is likely inconsequential. We found that the PCNF-BrA is an inconsistent branch. Maida and colleagues isolated the PCNF-BrA in their study but did not describe the prevalence [11]; we have not encountered this branch in our clinical practice. Additionally, there were no mentions of notable anconeus denervation nor functional deficits in the retrospective series of PCNF-BrEpi surgical denervation by Rose et al. and Dellon, and neither reports explicitly identified or isolated the PCNF-BrA as part of their surgical techniques [1,12]. In addition, there is a discrepancy regarding the motor innervation to the anconeus, as described by Maniglio et al., who describe the anconeus nerve (a direct branch off the trifurcation point of the distal radial nerve) to supply the motor innervation to the anconeus and do not mention or identify PCNF-BrA [13]. Provided these inconsistencies regarding the motor supply to the anconeus, and given the anconeus is an accessory muscle for elbow extension, the potential denervation by inadvertent PCNF-BrA injury likely has minimal-to-no functional consequence. Motor testing prior to ablation is another way to avoid inadvertent PCNF-BrA denervation.

The use of RFA for the treatment of chronic musculoskeletal pain is gaining favor. RFA has long been used for the management of cervical and lumbar facetogenic pain, knee pain, and hip pain. Sensory nerve denervation procedures such as RFA have been validated as a helpful alternative for joint pain that is refractory to intra-articular treatments such as corticosteroid injections and orthobiological treatments [14]. In the application of chronic joint pain treatment, RFA impairs the pain signal transmission from the pain generator through the thermocoagulation of the terminal articular sensory nerves. Along with the plantar fascia, the medial epicondyle of the humerus, and the greater trochanter, the lateral epicondyle of the humerus is one of the few osteotendinous structures that have described terminal sensory nerve innervation. The management of recalcitrant greater trochanter pain syndrome by terminal sensory nerve RFA has been described [15]. While still pending further validation, we propose that RFA of the PCNF-BrEpi should be considered in the treatment for recalcitrant LE. While orthobiological treatments usually require several months prior to reaching clinically significant therapeutic effects, RFA may result in more immediate pain relief and thereby facilitate meaningful engagement with physical therapy, earlier return to function, and activity/play [16,17]. A trial of RFA should be considered prior to surgical intervention since RFA carries a much lower risk for potential adverse effects compared to surgical treatments, as well as being less costly.

This novel treatment technique lends itself to the consideration of additional treatment variations. First, consistent with general approaches for sensory nerve RFA in interventional pain techniques, a prognostic block should be performed prior to the ablation. A negative prognostic block suggests either inadequate sonographic isolation or the presence of additional or alternative pain generators besides LE, which in either case would lead to poor response to RFA. If desired, a repeat prognostic block with sensory testing to improve nerve isolation can be considered. A second negative prognostic block should prompt further investigation for additional or alternative pain generators. Given the PCNF-BrEpi is a superficial nerve and easily accessible, alternative interventional techniques can be easily adapted. With regard to minimally invasive treatments, a trial of pulse RF or nerve hydrodissection may be considered prior to ablative treatments. Alternatively, chemodenervation of PCNF-BrEpi using 100% alcohol or phenol may also be suitable with only minimal modification of our technique. Additionally, as peripheral nerve stimulator (PNS) is gaining favor for the management of chronic pain conditions, the PCNF-BrEpi can be easily isolated for PNS treatment. Lastly, a referral for surgical neurectomy can also be considered, in which case the non-surgical interventionalist can provide vital information with pre-operative ultrasound nerve mapping.

## Conclusions

Our report describes the successful treatment of recalcitrant LE by US-guided PCNF-BrEpi RFA. This is the first report that illustrates the favorable clinical outcome of terminal sensory nerve RFA to the lateral epicondyle for the treatment of chronic tendinopathy. The technique we described can be utilized to perform other peripheral nerve interventional treatments with minimal modification as indicated clinically. Further investigation of this technique is necessary to validate RFA as well as its variations as a treatment option in recalcitrant LE.
